# The Control of Auxin Transport in Parasitic and Symbiotic Root–Microbe Interactions

**DOI:** 10.3390/plants4030606

**Published:** 2015-08-24

**Authors:** Jason Liang Pin Ng, Francine Perrine-Walker, Anton P. Wasson, Ulrike Mathesius

**Affiliations:** 1 Division of Plant Science, Research School of Biology, Australian National University, Linnaeus Way, Building 134, Canberra ACT 2601, Australia; E-Mail: Jason.Ng@anu.edu.au; 2 School of BioScience, The University of Melbourne, Parkville VIC 3010, Australia; E-Mail: marie.perrine@unimelb.edu.au; 3 CSIRO Agriculture, Canberra ACT 2601, Australia; E-Mail: Anton.Wasson@csiro.au

**Keywords:** auxin, nodulation, actinorhizal symbiosis, rhizobia, plant parasitic nematodes, mycorrhiza

## Abstract

Most field-grown plants are surrounded by microbes, especially from the soil. Some of these, including bacteria, fungi and nematodes, specifically manipulate the growth and development of their plant hosts, primarily for the formation of structures housing the microbes in roots. These developmental processes require the correct localization of the phytohormone auxin, which is involved in the control of cell division, cell enlargement, organ development and defense, and is thus a likely target for microbes that infect and invade plants. Some microbes have the ability to directly synthesize auxin. Others produce specific signals that indirectly alter the accumulation of auxin in the plant by altering auxin transport. This review highlights root–microbe interactions in which auxin transport is known to be targeted by symbionts and parasites to manipulate the development of their host root system. We include case studies for parasitic root–nematode interactions, mycorrhizal symbioses as well as nitrogen fixing symbioses in actinorhizal and legume hosts. The mechanisms to achieve auxin transport control that have been studied in model organisms include the induction of plant flavonoids that indirectly alter auxin transport and the direct targeting of auxin transporters by nematode effectors. In most cases, detailed mechanisms of auxin transport control remain unknown.

## 1. Introduction

The rhizosphere is colonized by a multitude of microbial species, many of which interact with plants. Indeed, the soil microbiome, which defines the specific microbial populations having close associations with a plant root system, has been termed the plant’s second genome [1], and is likely to expand the ability of the root system to respond to its environment. Root–microbe interactions can be mutualistic, parasitic or neutral, and often result in the formation of new root organs or alteration of the root architecture.

Three well-studied plant-microbe interactions are those between roots with mycorrhizal fungi, nitrogen fixing bacteria and parasitic nematodes. Mycorrhization is ancient and widespread and arises from host interaction with certain groups of fungi that confer enhanced uptake of nutrients, in particular phosphorus, and water to their hosts [2]. It leads to changes in root branching as well as re-arrangement of cortical cell development to house intra- and extracellular mycorrhizal fungi. Nodulation shows much greater host specificity and takes place in legumes and actinorhizal plants that form interactions with rhizobia and Frankia sp., respectively. These interactions lead to the formation of root nodules, which either arise through altered lateral root development or *de novo* nodule development from cortical cells [3]. An example of an important plant-parasitic interaction is the infection by root knot and cyst nematodes, causing millions of losses in crop production worldwide [4]. These parasitic nematodes cause the formation of feeding structures in the root that require changes in cell enlargement, division and differentiation [5].

Auxin, the first phytohormone to be identified [6], has been at the centre of every developmental process studied in plants [7]. Auxin represents a class of plant hormones of which the major form in higher plants studied thus far is indole-3-acetic acid (IAA). Since its discovery, auxin has been found in all land plants studied. Its activity has been associated with cell division, cell expansion and differentiation. Multiple auxin receptors exist in plants, that act both on the cell surface as well as intracellularly, highlighting the importance of auxin import into the cell. The most well-studied receptors belong to the TRANSPORT INHIBITOR RESPONSE1/AUXIN SIGNALING F-BOXs (TIR1/AFBs) family of F-box proteins, which localize to the nuclear membrane [8,9,10]. The binding of auxin to the TIR1/AFB receptor recruits the SKP, CULLIN, F-BOX-CONTAINING COMPLEX (SCF) that interacts with the former to produce the ubiquitin-ligase (E3) SCF^TIR1/AFB^ complex [11]. This complex initiates the removal of the AUXIN RESISTANT/INDOLE-3-ACETIC ACID (AUX/IAA) family of repressors from the cis elements of auxin responsive genes, and subsequent ubiquitination and degradation, thus activating auxin-induced responses in the cell [11,12]. The S-PHASE KINASE ASSOCIATED PROTEIN 2A (SKP2A) is another intracellular auxin receptor suggested to participate in cell cycle regulation [13,14]. SKP2A has been shown to directly bind auxin, and mutations in the putative auxin-binding pocket abolished auxin-SKP2A interaction [15]. A third auxin receptor, AUXIN BINDING PROTEIN1 (ABP1) was the first auxin receptor to be reported [16]. ABP1 is thought to be secreted into the cell wall, where it could function as the first line of sensing, before a signal is transduced into the nucleus [17]. However, a recent report showed that *abp1* mutants did not show any classical auxin-related developmental defects in Arabidopsis [18].

The range of plant growth and development programs involving auxin is extensive, encompassing the tropic responses, organ initiation, meristem maintenance and defence responses [19,20]. Considering its involvement in so many aspects of plant development, it is not surprising that auxin is one of the major targets for microbial manipulation. There are multiple lines of evidence showing auxin manipulation by microorganisms, including but not limited to nodulation, mycorrhization and nematode infection that form the focus of this review [21,22,23,24]. In these cases, auxin accumulation is often associated with the rapid proliferation of host cells during post-embryonic root organ formation. Changes in auxin dynamics can occur through biosynthesis, transport, conjugation or degradation [25]. In many instances it is difficult to uncouple these processes, as it is likely they act in concert to create a net change in auxin concentration and response at a given site. In fact, auxin is so crucial that many microbial species have acquired auxin biosynthesis capability, possibly through lateral gene transfer [26].

In this review, we discuss the role of auxin in symbiotic and parasitic plant microbe interactions, with a particular focus on auxin transport. Control of polar auxin transport has been shown to be of crucial importance for the generation of auxin gradients in the plant that are a prerequisite for initiating new organ development [27]. The next sections focus on our knowledge of auxin transport control in the plant, followed by evidence for the manipulation of these known auxin transport control points by micro-organisms, based on studies from selected well-studied model systems.

## 2. Auxin Transport Carriers Regulate Plant Development

**Figure 1:**
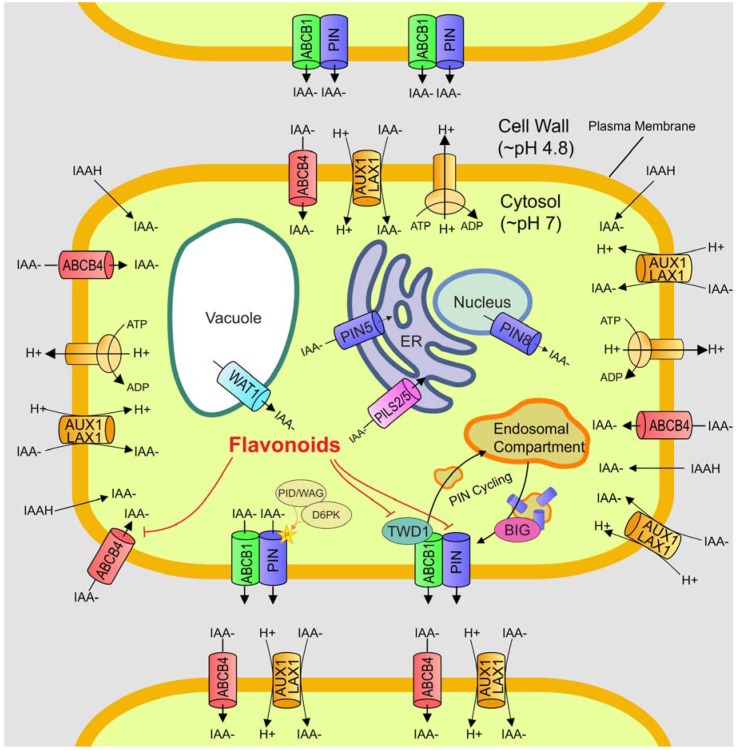
Molecular and chemiosmotic model of polar auxin transport. Molecular and chemiosmotic model of polar auxin transport. H^+^ATPase pumps maintain an acidic cell wall pH, which contrasts with the neutral pH of the cytosol. A proportion of the IAA in the acidic cell wall becomes protonated (IAAH), a state in which it can diffuse across the plasma membrane into the neutral cytosol, where is dissociates to a charged state (IAA^−^). Dissociated IAA is trapped in the cytosol unless actively transported. Dissociated IAA in the cell wall is actively transported into the cytosol by members of two classes of transporters. The AUX1/LAX1 family is proton dependent and reliant on the H^+^ATPase activity for the proton gradient. A member of the ABC superfamily of transporters, P-Glycoprotein 4 (PGP4)/ATP-Binding Cassette Subfamily B 4 (ABCB4), is an ATP-dependent auxin influx transporter, which can reverse the auxin efflux at high auxin concentrations [49]. Auxin influx is omnidirectional, but efflux confers polarity on the transport. Efflux is facilitated by active transport through the PIN family of transport proteins, which use membrane potential to transport auxin from the cytosol to the cell wall, and ABCB1 and ABCB19, other members of the ABC family which are ATP-dependent [50,51]. The PIN proteins confer polarity by localization to the acropetal side of the plasma membrane [36,52] via the cycling of PIN containing vesicles with endosomal compartments [53]. PIN protein activity can be modulated by phosphorylation through PID/WAG (PINOID/WAG) and D6PK (D6 PROTEIN KINASE) [54,55]. The ABCB efflux transporters form complexes with the PINs that enhance transport [56]. Other transporters are involved in intracellular transport. PIN8 has been found in the nuclear membrane [57] and PIN5 in the Endoplasmic Reticulum (ER) membrane [58]. Another family of auxin transporters, the PIN-LIKES (PILS) family has been identified; PILS2 and PILS5 have also been localized to the ER membrane [59]. Another atypical auxin transporter, WAT1, has been localized to the vacuolar membrane [60]. Flavonoids are auxin transport inhibitors [61] thought to disrupt the ABCB1 and ABCB4 proteins by binding the ATP binding cassette [50,62]. Flavonoids may also disrupt the complex between ABCB1 and TWD1 (TWISTED DWARF1) [63,64], affecting transport, and by binding BIG, a protein required for PIN cycling [65].

Auxin is primarily synthesized in young shoot tissues, from where it is transported to the roots, although other plant tissues are capable of producing auxin, too [28,29,30]. The transport of auxin can occur through two possible mechanisms: passive transport from source to sink tissues through the phloem and active transport across membrane barriers [31,32]. The latter, known as polar auxin transport (PAT), requires energy from ATP hydrolysis and is strictly regulated by auxin transport proteins (Figure 1). The phloem is capable of carrying auxin up to a speed of 7 cm/h [33], whereas PAT is usually a lot slower *i.e*., ~1 cm/h [34]. Subsequently, passive transport through the phloem is the likely route opted for long-distance transport [32], while the generation of local auxin gradients necessary for changes in plant development is achieved through PAT [35,36].

## 3. AUXIN RESISTANT (AUX)/LIKE-AUXIN RESISTANT (LAX) Proteins 

Auxin (IAA) is a weak acid (pKa = 4.75). Due to the slightly acidic apoplastic pH outside plant cells (pH ~5.5), most auxin remains in the protonated form and can diffuse across the hydrophobic plasma membrane [37,38,39]. There remains a proportion of deprotonated IAA^−^, which is actively taken up into the cell by auxin transport facilitators. In *Arabidopsis*, at least four auxin transporters are involved in auxin import, represented by *AUXIN-RESISTANT1* (*AUX1*) and *LIKE-AUX1* (*LAX1*), *LIKE-AUX2* (*LAX2)* and *LIKE-AUX3 (LAX3)* [40,41]. The AUX1 and LAX3 proteins are located at the plasma membrane (Figure 1). These importers function to translocate auxin into the cytoplasm [42,43,44,45] and this is presumed to involve proton co-transport based on similarity of AUX and LAX transporters to amino acid permeases [46]. AUX1 is dynamically remobilized between plasma membrane and internal compartments [47,48]. Movement between the Golgi apparatus and plasma membrane of AUX1 is independent of the trafficking regulator ADP Ribosylation Factor (ARF)-Guanine Nucleotide Exchange Factor (GEF) GNOM, and thus uses a distinct mechanism from the positional regulation of auxin exporters, such as PIN-FORMED 1 (PIN1), as discussed below [48].

## 4. PIN Proteins

Once inside the cell, the more alkaline (~pH 7) environment means auxin is present predominantly in the deprotonated form and has to be exported actively. The PIN-FORMED (PIN) family of auxin export proteins are essential for polar export of auxin (Figure 1), and eight members have been identified in *Arabidopsis* (PIN1-8), which differ in their location and function in plant development. Since then orthologues have been discovered in other plants, such as *Medicago truncatula*, *Populus trichocarpa*, *Solanum lycopersicum*, *Oryza sativa* and *Zea mays* [66,67,68,69,70,71]. The name owes its origin to the PINFORMED inflorescence phenotype observed in *Arabidopsis* mutant lacking PIN1, the primary auxin export facilitator. The PIN proteins can be further subdivided into full-length proteins (PIN1, 2, 3, 4, 7) and truncated forms (PIN5, 6, 8) [72]. Members of the former group are localized to the plasma membrane and their auxin export capabilities have been well-characterized in multiple heterologous systems [50,51,73]. Although auxin moves through the vascular tissue from shoot to root, it can be transported back up along the root cap cells and epidermis by PIN2 [74]. This reflux/fountain model provides an additional source of auxin in other high-demand areas, such as the elongation and the early differentiation zone of the root, where priming and initiation of lateral roots may occur. PIN8, a truncated PIN protein, has been found in the nuclear membrane in addition to the plasma membrane [57]. The authors postulated the organelle-localized PIN8 possibly functions to sequester auxin from the nucleus, thus controlling nuclear auxin signalling. Another atypical efflux carrier, PIN5, localizes to the endoplasmic reticulum membrane, presumably to control the translocation of auxin from the cytoplasm into the lumen of the endoplasmic reticulum [58].

## 5. Dynamic Repositioning of PIN Proteins during Developmental Responses

An important characteristic of the full-length PIN proteins, which will be repeatedly highlighted throughout this review, is their polar localization in the cell (Figure 1). This gives directionality to auxin flow and underlines the role of PINs in creating auxin maxima, such as during the initiation of new meristems like lateral roots, nodules and nematode feeding structures [23,27,75,76]. The asymmetric distribution of PIN proteins, especially PIN1, is modulated by actin filaments via rapid recycling of the proteins into endosomal vesicles and subsequent plasma membrane domain redirection [53]. The endosomal protein GNOM is involved in the polar recycling of PIN [77,78]. Drdova and colleagues [79] recently postulated that PIN recycling involves an exocyst complex, similar to those functioning in exocytosis in animals and yeasts, which acts downstream of GNOM. Phosphorylation of PIN by PINOID (PID) relocates the auxin transport protein to the basipetal side of root cells through a GNOM-independent pathway. This process is negatively regulated by phosphatase 2A (PP2A) [54]. Hence, this mechanism allows plants to respond quickly by channelling auxin into the appropriate tissues upon receiving external stimuli. Besides PID, phosphorylation of PIN proteins is also achieved via another kinase belonging to the AGCVIII family of kinases in Arabidopsis, *i.e.* D6 PROTEIN KINASE (D6PK) [55]. Both PID and D6PK kinases serve non-redundant functions by phosphorylating different sets of residues located at the cytoplasmic loop of PIN proteins. Phosphorylation of PIN residues activates the proteins and this adds another layer of regulation that can potentially be targeted by microorganisms.

## 6. P-Glycoprotein/ATP-Binding Cassette Proteins

A second group of transporters involved in auxin export is the family of P-GLYCOPROTEINS/ATP-BINDING CASSETTE SUBFAMILY B (ABCB) transporters (Figure 1). However, unlike PINs, their arrangement at the plasma membrane is usually not polar [72]. PGP proteins function in non-polar efflux of auxin. PGP1 and PGP19/MDR1 both interact and form complexes with the immunophilin-like protein TWISTED DWARF1 (TWD1). Disruption of this interaction affects plant development, including epinastic growth and reduced inflorescence size [50,80]. At least one member of the PGP family, PGP4, exhibits auxin import activity in addition to its export capability in certain cells [49,62,81]. PGP4 appears to be an inducible exporter. Under low IAA conditions, it operates as an auxin importer whereas it reverts to being an exporter when IAA concentrations increase. Direct evidence suggests the primary role of PGP is to restrict auxin flow in the main transport streams and to prevent reflux of auxin. This is observed in small cells where PGPs and auxin occur at high levels [56,82,83]. Models for PIN-PGP crosstalk have been drawn. In cases where PGP co-localizes with PIN, they work in tandem to direct auxin translocation into specific cells. Nevertheless, their mostly non-polar distribution in cells means that PGPs regulate the effective cellular auxin concentration [49,56,83,84].

## 7. PILS Proteins

More recently, a novel group of intracellular auxin transport regulators was identified through *in-silico* analysis. The *PIN-LIKES* (*PILS*) family of genes encode proteins with similar topology to the PIN proteins, *i.e*., they contain a predicted central hydrophilic loop flanked by five transmembrane domains on both sides [85,86]. In addition, PILS contain the InterPro auxin carrier domain. Despite sharing similar topology to PINs, the amino acid sequence between these two families of proteins share low similarity (<20%) [85]. Nevertheless, Feraru and colleagues analyzed the diversification of PILS and found that they are generally conserved throughout the plant lineage [59]. Putative PILS members in other model organisms were also highlighted. PILS are evolutionarily older because PILS are present in ancient plants, such as the unicellular algae *Ostreococcus tauri* and *Chlamydomonas reinhardtii*, where PINs are absent [58,85]. The importance of intracellular auxin homeostasis is given further weight by the discovery of another tonoplast-localized auxin carrier, WALLS ARE THIN1 (WAT1), which is a plant specific protein and is also the first vacuolar auxin transport protein to be identified (Figure 1) [60].

Intercellular auxin transport is controlled by the three groups of auxin carriers mentioned above, *i.e*., AUX1/LAX, PIN and PGP/ABCB. This clearly demonstrates auxin transport as a complex system, and one of many plant-evolved mechanisms controlling a plethora of developmental responses. Based on current knowledge in a few model systems, the roles of auxin transport proteins during plant-microbe interactions are evident but genetic and biochemical evidence for the involvement of most of the mentioned auxin transporters is still incomplete. Furthermore, it remains to be seen whether other carrier-carrier interaction exists apart from the currently known PIN1-PGP19 coupling. It is also worthwhile to note that research has so far focused on the transport of the active auxin IAA. Nevertheless, transport of other auxin metabolites, such as IBA [87,88], should be investigated in the future. Liu and colleagues demonstrated a lower transport occurrence of IBA than IAA in *Arabidopsis* but this could differ in other plant species [87].

## 8. Flavonoids are Natural Auxin Transport Modulators

Flavonoids form one of the best-studied groups of secondary metabolites, and are products of the phenylpropanoid pathway. Their roles are diverse, ranging from pathogen defence, UV protection, symbiosis, allelopathy, quorum sensing, as well as giving colour to flowers and fruits [89,90,91]. Flavonoids also modulate plant development by acting as natural auxin transport inhibitors [61], and flavonoid biosynthesis mutants typically show altered auxin transport characteristics.

Not all flavonoids have been shown to exhibit auxin transport inhibition. The flavonol subclass in particular, such as kaempferol and quercetin, show the strongest inhibitory activity [61,92]. Flavonoid over-accumulating mutants show decreased auxin transport capacity whereas the opposite has been observed for flavonoid-deficient mutants [93,94,95]. Flavonols inhibit auxin transport by competing with synthetic auxin transport inhibitors (including 1-naphthylphthalamic acid, NPA, and 2,3,5-triiodobenzoic acid, TIBA) for plasma membrane and microsomal binding sites [61,96,97]. *Arabidopsis* mutants lacking flavonoids show altered PIN expression and localization [94]. The PID/WAG kinases regulate PIN subcellular localization and are flavonoid-sensitive [98,99,100,101]. As mentioned above, PID and PP2A act antagonistically to regulate polar localization of PIN1 (Figure 1) [54]. Hence, flavonoids may indirectly modulate PAT via inhibition of PID/WAG. Flavonoids were also able to partially restore asymmetric PIN1 sub-cellular localization and lateral redirection of auxin stream in the *pin2/eir1* mutant [102].

Flavonoids may also act on PGP-mediated auxin transport. The binding of flavonols to mammalian and plant PGP transporters inhibits their activity both *in vivo* and in heterologous systems [50,62,93,103,104,105]. Mammalian PGPs are regulated through phosphorylation, and flavonoids were found to disrupt ATPase activity through phosphorylation and allosteric binding [106]. Since PGPs are highly conserved between species, flavonoids might regulate plant PGPs via phosphorylation [96,107,108]. Moreover, the flavonols kaempferol and quercetin have been demonstrated to disrupt the binding between PGP1 and its activator TWD1 (Figure 1) [63,64].

Flavonoids and auxin seem to be closely associated in several developmental responses. Accumulation of auxin and expression of auxin transport carriers are accompanied by an increase in flavonoid concentration [62,105,109,110]. Flavonoid aglycones are present in low concentrations inside the cells [111]. The majority of flavonoids exist as glycosides stored in vacuoles. The active form of flavonoids involved in auxin transport inhibition is still questionable and while it was thought that flavonoid glycosides are mainly inactive, recently a glycoside of kaempferol was shown to inhibit auxin transport in *Arabidopsis* shoots [112]. In the *Arabidopsis* mutant *rol1-2* (*repressor of lrx1*), flavonols specifically inhibit cellular export of naphthalene acetic acid (NAA) but not IAA, suggesting a possible developmental or tissue-specific mode of action of flavonols [113]. Despite their multiple roles in controlling auxin transport, complete flavonoid deficiency in *Arabidopsis* does not prevent normal plant development. Plant organogenesis, such as lateral root formation, even though it is strictly dependent on auxin transport control, still occurs in the absence of flavonoids [93,114,115]. Interestingly, flavonoids accumulate specifically during interactions with bacteria, nematodes and fungi [89,116,117,118], where they could be ideal mediators between the perception of microbial signals and changes in plant development. The function of flavonoids in plant-microbe interactions, in particular their effect on auxin transport, is discussed in the following sections. We begin with the interactions of most land plants with parasitic nematodes, and then focus on mutualistic interactions, starting with the most ancient mycorrhizal associations, and then discussing different evolutionary transitions in the symbiosis between plants and nitrogen fixing bacteria [119].

## 9. Auxin Transport Regulation during Feeding Site Establishment by Plant Parasitic Nematodes

Plant parasitic nematodes establish feeding sites on a wide range of host plants, most likely through injection of parasite effectors into host cells [120]. Cyst nematodes (*Heterodera* ssp. and *Globodera* ssp.) trigger the development of cysts in their hosts’ roots that are characterized by the formation of a syncytium following cell wall dissolution and fusion of multiple cells in the procambium, pericycle or cortex [5]. Root knot nematodes (*Meloidogyne* ssp.) stimulate endoreduplication in vascular parenchyma cells, which results in the formation of multinucleate, so-called “giant cells” [5]. The expansion of the giant cells is often accompanied by extensive divisions of surrounding pericycle and cortical cells, and this leads to the formation of root galls. The initiation of pericycle cell divisions in the galls often stimulates the emergence of lateral roots at the base of a gall [121,122]. So far it is largely unknown which nematode effectors cause feeding structure formation.

As in other root organs, auxin was detected in developing feeding structures [123,124], where it is likely involved in the regulation of cell division and differentiation of root galls, in the regulation of cell wall loosening during syncytium development, and in the formation of new vascular tissue [5,125,126]. This has been supported by the detection of increased expression of a number of genes encoding auxin response and auxin transport proteins [125,126,127,128,129]. In cyst nematode-infected roots, auxin-inducible cell cycle genes were found to be activated [130], while auxin-repressed genes were down-regulated [131]. Increased expression of auxin reporters was localized in early dividing cells in developing galls [117,121,132] and syncytia [23,121,132]. The requirement for auxin in feeding site establishment was confirmed by the finding that auxin-insensitive *Arabidopsis* and tomato mutants show defects in feeding cell establishment [122].

The increased auxin content in galls could be a direct result of auxin synthesis by nematodes or of indirect manipulation of auxin transport or response in the host, triggered by nematode secretions. Whether auxin is synthesized by parasitic nematodes has been questioned [122]. Instead, there is increasing evidence that the effectors of parasitic nematodes cause changes in auxin metabolism and transport in the host to redirect auxin into the growing feeding structure.

For example, *Arabidopsis* mutants defective in auxin transporter-encoding genes were impaired in syncytium development, and treatment of plants with synthetic auxin transport inhibitors interfered with syncytium development, suggesting that plant parasitic nematodes cause auxin accumulation by inhibiting polar auxin transport [122]. Similar to nodule development, this inhibition of auxin transport may be mediated by flavonoids, which accumulate in developing galls [117] and syncytia [133]. However, flavonoid-deficient *Arabidopsis* mutants were still able to form feeding sites when infected with cyst nematodes [133], and *M. truncatula* hairy roots in which the flavonoid pathway was silenced by RNA interference were still able to form giant cells after infection with root knot nematodes, although the galls were found to be smaller [115]. Interestingly, flavonoid synthesis is activated by the transcription factor WRKY23 in *Arabidopsis*, which was initially isolated as a nematode-induced gene [109]. It is likely that flavonoids modulate auxin transport in feeding sites but that they are not essential for their initiation.

Another mechanism for auxin relocation into a developing feeding structure includes increased auxin import through the AUX/LAX family of auxin import proteins, similar to auxin transport into cortical cells positioned in front of lateral root primordia through LAX3 during lateral root emergence [43,134] (Table 1). This is supported by strong expression of the gene encoding the auxin importer AUX1 in developing feeding structures of both cyst and root knot nematodes [135], and an increase in *AUX4* expression in *Arabidopsis* infected with *M. incognita* [136]. More recently, the cyst nematode effector HS19C07 from *Heterodera schachtii* was demonstrated to interact directly with the LAX3 auxin importer from *Arabidopsis* [137], and double and quadruple mutants of *aux1/lax3* and *aux1/lax1/lax2/lax3* displayed less infections by *H. schachtii*. At the same time as increasing auxin import into developing feeding structures, there is evidence for redirection of auxin transport into syncytia through relocation of PIN proteins. In *Arabidopsis*, a study utilizing *pin* mutants and *PIN* reporters showed that cyst formation requires down-regulation of *PIN1* at the initiation stage, probably to cause auxin accumulation. This is followed by lateral auxin transport by PIN3 to redirect auxin flow into a developing feeding site [138], similar to the PIN3-dependent auxin reflux from endodermal into lateral root founder cells [139]. These studies suggest that plant parasitic nematodes target both auxin import proteins to channel auxin into growing feeding structures, and relocating auxin export proteins laterally to expand cell divisions and cell fusions in feeding structures. However, so far no direct measurements of auxin transport have been done in nematode-infected roots, and this would be important to do in the future. The use of fluorescently labelled auxin analogs [140] would be particularly exciting for demonstrating local, and lateral auxin transport changes in developing feeding structures. In addition, it will be important to integrate the action of auxin in feeding site establishment with the role of cytokinin, as it is likely that both hormones act together in feeding site formation. For example, auxin and cytokinin responses overlap in developing phloem surrounding developing feeding cells [126].

**Table 1:** Summary of auxin transport proteins implicated in root–microbe interactions.

Gene/Protein	Host Organism	Type of Interaction	Role/Phenotype	Reference
AtAUX1	*Arabidopsis thaliana*	*Heterodera schachtii*	- AUX1 expression was localized to feeding sites	[135]
AtPIN1 AtPIN2	*Arabidopsis thaliana*	*Heterodera schachtii*	- Reduced *H. schachtii* development in infected roots of *pin1/tgg1* and *pin2* mutants	[122]
AtAUX1 AtLAX1 AtLAX2 AtLAX3	*Arabidopsis thaliana*	*Heterodera schachtii*	- AtLAX3 interacts with cyst nematode effector Hs19C07	[137]
			*- aux1lax1* and *aux1lax1lax2lax3* mutants show reduced infection by *H. schachtii*	
AtPIN1 AtPIN2 AtPIN3 AtPIN4 AtPIN7	*Arabidopsis thaliana*	*Heterodera schachtii*	- *PIN1, 2, 3, 4* genes are expressed in developing feeding structures, but at different times	[138]
			- PIN3 and PIN4 are redirected to lateral sides of feeding cells	
			- Mutants of all five *PIN* genes show reduced infection	
AtLAX3 AtAUX1	*Arabidopsis thaliana*	*Meloidogyne incognita*	- AtLAX3 and AtAUX1 expression induced in developing root galls	[136]
PtaPIN2, 4,9,12	Poplar (*Populus tremula × Populus alba*)	*Laccaria bicolor* (Ectomycorrhiza)	- Gene expression upregulated during early interaction	[21]
PtaAUX6	Poplar	*Laccaria bicolor* (Ectomycorrhiza)	- Gene expression upregulated during early interaction	[21]
AtPIN2	*Arabidopsis thaliana* (nonhost)	*Laccaria bicolor* (Ectomycorrhiza)	- LR induction reduced by 40% in *Atpin2* mutant	[21]
AtAUX1	*Arabidopsis thaliana* (nonhost)	*Tuber borchii* (Ectomychorizza)	- Reduced inhibition of primary root development in *Ataux1-7* mutant compared to WT	[141]
CgPIN1	*Casuarina glauca*	*Frankia* inoculation	- Localized to uninfected cells adjacent to *Frankia*-infected cells	[142]
			- Exports IAA into *Frankia*-infected cells	
CgAUX1	*Casuarina glauca*	*Frankia* inoculation	- Localized to *Frankia-*infected cells	[143]
			- Likely imports IAA into *Frankia*-infected cells	
DtAUX1	*Discaria* *trinervis*	*Frankia* inoculation	-Localized to nodule meristem	[144]
MtPIN2	*Medicago truncatula*	*Sinorhizobium meliloti* inoculation	- Expressed in peripheral vasculature in early nodule primordium	[145]
			- Expressed at the base of mature nodule	
			- Knockdown of *MtPIN2* reduced nodulation	
MtPIN3	*Medicago truncatula*	*Sinorhizobium meliloti* inoculation	- Knockdown of MtPIN3 reduced nodulation	[107]
MtPIN4	*Medicago truncatula*	*Sinorhizobium meliloti* inoculation	- Knockdown of MtPIN4 reduced nodulation	[43]
		*Sinorhizobium meliloti* nod factor treatment	- Increased expression after nod factor treatment	[146]
MtPIN10	*Medicago truncatula*	*Sinorhizobium meliloti* nod factor treatment	- Increased expression after nod factor treatment	[147]
MtLAX1-3	*Medicago truncatula*	*Sinorhizobium meliloti* inoculation	- Transcripts are localized to early dividing cells and to cells near the vasculature of early nodule primordium	[148]
LjABCB1	*Lotus japonicus*	*Mesorhizobium loti* inoculation	- Localized to uninfected cells adjacent to Rhizobia-infected cells	[149]
			- Exports IAA from uninfected cells into adjacent Rhizobia-infected cells	

## 10. Auxin Changes during Mycorrhizal Interactions

The symbiotic interaction with mycorrhizal fungi is evolutionarily ancient and occurs in more than 80% of all land plants. Mycorrhizal fungi stimulate and modify root growth and aid in phosphorus and other nutrient uptake from the soil [150]. In return, the plant host provides carbon metabolites and serves as a shelter for the survival and reproduction of the symbiont. Like for all fungi, mycorrhiza classification is difficult, especially with the heterogeneity of this group where members are spread over diverse fungal taxa [2]. They can be broadly classified into two groups, namely the ectomycorrhizae and endomycorrhizae. The terminology arises from the symbiont’s ability to penetrate plant cell walls during the infection process [151]. Ectomycorrhizae surround the root tip with a thick mantle of hyphae and more hyphal structures grow inwards but only intercellularly, forming the Hartig net, which is a latticework of hyphae surrounding epidermal and cortical cells. Infection by this class of fungi usually results in root tip bifurcation and arrest of root growth. Endomycorrhizal fungi, such as arbuscular mycorrhizae (AM), develop hyphae from a spore, producing a hyphopodium on the root epidermis. The hyphae penetrate inwards intra- and intercellularly until they reach the inner cortical cells, where they finally form tree-like protrusions inside the cells, called arbuscules [2].

The role of auxin during mycorrhizal formation is not surprising. Mycorrhizal fungi often stimulate root elongation and lateral root formation, probably as a strategy to increase infection area. Lateral root formation requires spatially and temporally controlled changes in auxin transport and response [152], and external auxin application or elevated auxin biosynthesis is sufficient to cause lateral root outgrowth [35,153]. Furthermore, auxin transport inhibitors cause arrest of lateral root development through inhibition of basipetal auxin transport in the root [154]. Polar auxin transport and activation of the auxin signalling pathway are implicated during lateral root founder cell specification, which occurs within a narrow auxin minimum zone [155]. In general, auxin homeostasis controls every stage of lateral root formation [35]. Since plant-mycorrhizal interactions alter root branching, the involvement of auxin in this process has been postulated [22,101].

### 10.1. Ectomycorrhizal Symbioses

A requirement for basipetal auxin transport during ectomycorrhizal-induced lateral root proliferation was proposed [21]. The authors identified 16 auxin-related transcriptional changes during the interaction between *Laccaria bicolor* and poplar, including genes encoding components of polar auxin transport (*PtaPIN2*, *PtaPIN4*, *PtaPIN9*, *PtaPIN12*) (Table 1). The putative auxin exporter *PtaPIN9*, encoding a protein orthologous to the *Arabidopsis* PIN2 protein involved in basipetal auxin transport at the root apex, was upregulated during early root–fungus interaction. Consistent with this, induction of lateral root formation in *Arabidopsis* *pin2* roots reduced by ~40% compared to WT, in the presence of *L*. *bicolor*. This reduction is contributed exclusively by basipetal auxin transport because the quadruple mutant *pin2,3,4,7* did not show additional reduction. Similar results were obtained when *Arabidopsis* interacted with truffle fungi (*Tuber borchii* and *Tuber* *melanosporum*) [141]. The authors could mimic the primary root shortening and lateral root branching phenotypes in the non-host *Arabidopsis* and host *Cistus incanus*. These effects, however, are contributed by auxin and ethylene collectively, because auxin treatment by itself could only phenocopy primary root shortening. Indeed, the auxin transport mutant *aux1-7* was insensitive to primary root growth arrest, illustrating auxin transport as an important component in mycorrhiza-mediated root architecture modulation [141]. Moreover, general inhibition of polar auxin transport by NPA treatment decreased lateral root numbers [21].

Ectomycorrhizal fungi have long been known to synthesize and secrete auxin [156]. Mutant hyphae of *Hebeloma cylindrosporum*, which overproduce auxin result in up to five-fold more mycorrhizas relative to a wild-type mycelium [157,158]. During the *Picea abies*–*Laccaria bicolor* symbiosis, application of the auxin transport inhibitor TIBA resulted in defective Hartig net formation, less intercellular fungal colonization and reduced root branching in fungal-infected roots, suggesting that auxin transport control is an important mechanism involved in ectomycorrhiza-mediated root architecture modulation [159]. More recently, data from high-throughput sequencing revealed a down-regulation of several auxin transport and auxin-related transcription factor genes in mature oak ectomycorrhizas, suggesting that auxin transport and signalling play an important role during early symbiosis [160]. 

Two *Laccaria bicolor*—regulated nodulin-like ESTs homologous to *M*. *truncatula* nodulin genes—were identified in *Pinus sylvestris* [161,162]. Co-inoculation of ectomycorrhiza and the auxin transport inhibitor TIBA resulted in higher expression of these two genes, suggesting a requirement for auxin transport inhibition for the induction of these nodulin-like genes. Moreover, it has been reported that NPA treatment results in root bifurcation comparable to that induced by ectomycorrhiza [163,164]. It has been postulated that NPA treatment simulates an auxin accumulation end result during ectomycorrhizal symbiosis, where the tight Hartig net around epidermal and cortical cells prevents auxin recycling and subsequently concentrates auxin locally [164].

### 10.2. Endomycorrhizal Symbioses

It is possible that AM fungi produce auxin in addition to Myc factors, the crucial signal molecules mediating mycorrhizal signalling [165]. This might explain the symbiosis (SYM) pathway-independent induction of lateral roots in rice, whereas in nodulating legumes the common SYM pathway is involved in LR induction [166]. Sirrenberg and colleagues [167] showed that the AM-like fungus *Piriformospora indica* is capable of producing auxin in liquid culture, while other AM species, like *Rhizophagus irregularis* are lacking typical auxin biosynthesis genes [168]. AM fungi were also shown to alter auxin concentrations and auxin synthesis in their host plants, although the mechanism for this effect remains unknown and is highly dependent on the host species [169,170,171]. Myc factor application to *Medicago truncatula* roots induced lateral roots, although it is unclear whether this was due to increased auxin accumulation in the root [172]. Transcriptome analysis in AM-infected rice roots showed root-type specific accumulation of auxin-induced genes, as well as changes in auxin transporter-encoding genes [173]. Interestingly, the changes in auxin response gene expression seen during AM infection agree with the role of those genes in lateral root formation in rice [173]. However, it is also possible that auxin responses have a role in the infection process as localisation of auxin responses in AM-infected rice roots showed high auxin sensitivity in arbuscule-containing cells [174], although this was not found during AM formation in *Tropaeolum majus* [169]. The induction of auxin responsive genes during AM symbiosis in rice is supported by the finding that down-regulation of auxin receptors through modification of microRNA393 reduced mycorrhization in rice [174].

Functional support for the role of auxin transporters in AM symbioses in tomato was reported in a study by Hanlon and colleagues [175]. An auxin-resistant mutant, *diageotropica* (*dgt*) caused hyphal growth away from cultured tomato roots, resulting in termination of infection. By contrast, a hyperactive polar auxin transport mutant, *polycotyledon* (*pct*) had faster and more extensive cultured root colonization by the AM fungus *Glomus* (*Rhizophagus*) *intraradices* compared to WT. Interestingly, a previous study by Xie and colleagues found that TIBA treatment increased colonization rate of *Lablab purpureus* with an AM fungus [176]. This is also accompanied by increased appressorium formation [177]. The ability of Nod factors to stimulate mycorrhization might be attributed to polar auxin transport inhibition similar to indeterminate nodulation [176]. Localization studies of auxin transporters in AM systems remain sparse. A study of AM-infected *Casuarina glauca* roots did not find any evidence of enhanced *CgAUX1* expression in infected regions [178]. While the potential signals used to control auxin transport during AM formation is unknown, flavonoids are unlikely to be candidates because flavonoid-deficient plants can still form mycorrhizae [179]. Overall, while there is strong suggestion for the modification of auxin responses and transport during mycorrhizal infection of hosts, detailed localization and functional characterization of auxin transport proteins and their regulators is still lacking.

## 11. Auxin Transport Control in Nitrogen Fixing Symbioses

### 11.1. Actinorhizal Symbioses

Actinorhizal symbioses are formed between members of eight plant families, called actinorhizal plants, and filamentous actinobacteria, of which the genus *Frankia* has been studied most extensively [180]. Actinorhizal symbioses involve either intra- or intercellular infection by the symbiont, and the formation of a nodule that resembles a modified lateral root. Auxin accumulation in these nodules has been shown in multiple studies, and could be involved in cell wall modification during infection thread formation or cell hypertrophy, metabolism or regulation of gene expression in infected cells [119,143].

*Frankia* has been shown to synthesize auxin in culture, including the auxin phenyl acetic acid (PAA) (e.g., [181,182]). PAA is also synthesized by plants, but might have roles beyond auxin action in plants as it is a constituent of hopanoid lipids required for the formation of vesicle envelopes of *Frankia* during infection [147,182]. While auxin accumulation has been demonstrated in infected cells of actinorhizal nodules, it remains unclear to what extent this is contributed by the symbiont as opposed to the host. In *Frankia*-infected nodules of *Casuarina glauca*, auxin was detected in all infected cells irrespective of their distance to the host nodule cells, suggesting that at least some of the auxin in infected cells is contributed by the symbiont [142]. In addition, all major auxin synthesis genes are encoded by *Frankia* and their expression is enhanced in response to N starvation [142]. However, there is growing evidence that redirection of auxin transport within actinorhizal nodules is also occurring as a result of changes in auxin transporter localization (Table 1).

**Figure 2:**
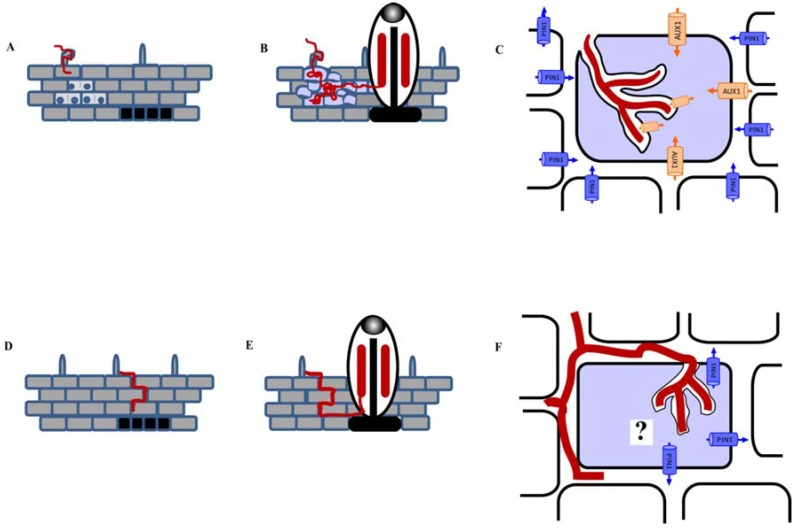
The role of auxin in two different actinorhizal nodulated plants *Casuarina* *glauca* and *Discaria* *trinervis*. Schematic model proposing the role of auxin during actinorhizal nodule development in the she-oak *C*. *glauca* (**A**–**C**) and the actinorhizal shrub belonging to the *Rhamnaceae* family, *D*. *trinervis* (**D**–**F**) when interacting with *Frankia* sp. In *C. glauca*, *Frankia* causes root hair curling and infects the curled *C. glauca* root hair. This also triggers cells in the cortical region of the root to undergo mitotic division while cells in the pericycle (black) are also undergoing cell division (**A**); (**B**) Infected cells in the cortical region form a prenodule structure that is composed of infected and uninfected cells (light blue). *Frankia* hyphae from the prenodule infect the cortical cells located at the base of the nodule primordium and progressively invade the cortex of the nodule lobe via intracellular infection. CgAUX1 is involved in curled root hair infection, infection of the prenodule and nodule primodium [151]; In (**C**) a model was proposed for how two auxin transporters, CgAUX1 (auxin influx, orange) and a putative CgPIN1 (auxin efflux, blue), contributed to the auxin accumulation in *Frankia*-infected cortical cell (light blue). Using computer simulations with microscopic multiphoton confocal images, it was demonstrated that the expression of CgAUX1 in the *Frankia*-infected cells and the putative CgPIN1 in neighbouring uninfected cells could contribute to the accumulation of auxin in *Frankia*-infected cells. In *D. trinervis*, *Frankia* infects the root tissue intercellularly while cells (black) in the pericycle layer undergo division (**D**) to subsequently form the actinorhizal nodule primordium shown in (**E**). In (**F**), work by Imanishi and colleagues [144] demonstrated that DtAUX1 and putative DtPIN1 do not behave in the same manner to cause auxin accumulation in *Frankia*- infected cells in the *D. trinervis* actinorhizal nodule. DtAUX1 expression was only observed in the meristematic cells of nodules. PAA was detected only in *Frankia*-infected cell (light blue). Such enlarged cells appeared to expressed putative DtPIN1. It is proposed that other auxin transporters may be needed to cause auxin accumulation in such cells (indicated by “?”). Anatomy of the nodules for both actinorhizal plants originate from the pericycle layer shown in **B** and **E**. Nodule apical meristems are in grey and the tissues colonized by *Frankia* are in red (in **B** and **E**). The intracellular and intercellular modes of infection by *Frankia* (in red) are depicted in **A**–**C** and **D**–**F**, respectively. Figure modified from [104].

For example, during the symbiosis between the actinorhizal tree *Casuarina* *glauca* and *Frankia*, expression of the auxin importer *CgAUX1* was associated with *Frankia*-infected cells throughout the infection process, and treatment with the auxin influx inhibitor 1-naphthoxyacetic acid (1-NOA) severely reduced nodule numbers, underlining the importance of auxin influx in actinorhizal nodulation [143]. It was later discovered that the *C*. *glauca* *PIN1-like* gene was selectively localized to uninfected cells [142]. Coupled with computer simulations, the authors suggested that CgAUX1 and CgPIN1-like are arranged in this manner to direct auxin accumulation in *Frankia*-infected cells (Figure 2).

Interestingly, recent work by Imanishi and colleagues [144] demonstrated that auxin accumulation also played a role in actinorhizal nodule development in a shrub of the *Rhamnaceae* family, *Discaria trinervis*. Similarly to *C. glauca*, auxin accumulation was detected in *Frankia*-infected cells in *D. trinervis* by immunolocalization of PAA [144]. In addition, 1-NOA also perturbed nodule formation in *D. trinervis.* However in contrast to the *C. glauca* model system, it was found that the endogenous *DtAUX1* gene expressed only in the meristematic region of actinorhizal nodule and not in the *D. trinervis* infected cells [144]. This was consistent with the location of the auxin response marker *DR5:VENUS* in the meristem of *Frankia*-treated transgenic *D. trinervis* plants [144]. Furthermore, PIN1 immunolocalization studies detected a DtPIN1-like efflux transporter on the plasma membrane of hypertrophied cortical cells infected by *Frankia* (Figure 2; [144]). This suggests that the distribution of auxin transporters in *Frankia*-infected *D. trinervis* is different to that observed in *C. glauca* (Figure 2). *In silico* studies proposed that DtAUX1 and a DtPIN1-like activity were insufficient to explain the auxin accumulation and perception in *D. trinervis* nodules [144]. In *D. trinervis*, the mode of infection by *Frankia* is intercellular as opposed to the intracellular mode of infection observed in *C. glauca* (Figure 2; [183]). Thus the role of auxin and its transport during actinorhizal symbiosis is likely to be different in intracellular- infected *C. glauca* and intercellular-infected *D. trinervis* [144].

Isoflavonoids have the capability to control auxin transport, and transcript profiling during *C*. *glauca*-*Frankia* association suggest a role for isoflavonoids during nodulation in this system [184]. In addition, silencing of the first enzyme of the flavonoid pathway, chalcone synthase, in *C. glauca* significantly reduced nodulation [185]. However, whether isoflavonoids play a role in actinorhizal nodulation through auxin transport control, or whether they are involved in activation of the bacterial symbiont in the soil, remains to be tested.

### 11.2. Legume-Rhizobium Symbioses

Nitrogen fixation occurs in legumes as a result of the interaction between legume hosts and a group of α-proteobacteria collectively known as rhizobia. Some legumes can also form nodules in symbiosis with β-proteobacteria, e.g., *Burkholderia* spp. [3]. This interaction culminates in the formation of root, and sometimes stem nodules, in which nitrogen fixation takes place. Nodulation is initiated by flavonoid exudates from roots of legume hosts. Flavonoids transcriptionally activate Nod factor (lipochitin oligosaccharide) synthesis in their specific *Rhizobium* symbionts [186,187]. Nod factor perception by the host plant in turn activates a cascade of signalling events, which results in nodule organogenesis originating from cell divisions in the pericycle, endodermis and cortical layers [188,189]. However, in some legumes nodules may also form from a modified lateral root, similar to actinorhizal nodules [3,190]. Rhizobia can enter the host through crack entry at points of lateral root emergence or via an infection thread. Once rhizobia have entered cells of the nodule primordium, they differentiate into bacteroids inside symbiosomes, which are highly specialized organelle-like structures optimized for nitrogen-fixing activity.

Several forms of nodules have been described. Indeterminate nodules can be found in many temperate legumes, such as white clover (*Trifolium repens*), pea (*Pisum sativum*) and barrel medic (*Medicago truncatula*). Nodules formed on these plants originate from pericycle, endodermis and inner cortical cell divisions. They are characterized by a persistent meristem, resulting in an elongated nodule form [24,188]. On the other hand, determinate nodules do not have a persistent meristem and thus they are defined by a globular shape. Nodules of this type are initiated by middle and outer cortical cell divisions, which subsequently fuse with divisions in the pericycle. Such nodules are seen on (sub)tropical legumes, for instance common bean (*Phaseolus vulgaris*), soybean (*Glycine max*) and birdsfoot trefoil (*Lotus japonicus*). Indeterminate and determinate nodules represent the best-studied forms of nodulation, although many other forms of nodulation have been identified [191].

As for other symbiotic bacteria, auxin is synthesized by rhizobia (e.g., [192]) and might directly contribute to successful nodulation. For example, rhizobia strains overproducing IAA increased nodule numbers in *Medicago truncatula* and *M. sativa,* whereas this effect was not seen in *Phaseolus vulgaris*, which forms determinate nodules [193]. However, auxin response and auxin transport changes have been found even in response to purified Nod factors from rhizobia [194,195], demonstrating that auxin transport and accumulation are changed in the root in response to the Nod factor-signaling pathway.

Interestingly, formation of uninfected nodule-like structures can be mimicked by external application of synthetic auxin transport inhibitors to the roots, suggesting that polar auxin transport inhibition is part of rhizobia’s toolbox for making a nodule [196,197]. Induction of pseudo-nodules by synthetic auxin transport inhibitors in *M. truncatula* mutants defective in early Nod factor signalling suggested that auxin transport inhibition occurs downstream of NFP, LYK3, DMI1, DMI2, DMI3, NIN, and RIT1 action [198]. Pseudonodulation has also been demonstrated in *Medicago sativa*, tobacco, *Alnus glutinosa* and *P*. *sativum* [196,199,200,201]. These nodule-like structures usually form through cortical cell division and subsequent expansion, with a final globular structure usually containing lobes. However, they do share some features with *Rhizobium*-induced nodules, such as a central tissue, (pseudo)nodule cortex and an equivalent tissue of the nodule parenchyma, as well as expression of some nodulation genes [196,198]. In contrast, synthetic auxin transport inhibitor treatments to *L. japonicus* roots did not induce pseudonodule formation [202]. This suggests a difference in the requirement for auxin transport during indeterminate and determinate nodulation programs. The ability of host plants, at least those forming indeterminate nodules, to form nodule-like structures without interacting with *Rhizobium*-derived signals, such as through exogenous auxin transport inhibitor treatments, suggests that formation of nodules is host-autonomous.

Redirection of polar auxin transport is likely required for accumulation of auxin in developing nodules. Changes in auxin accumulation have mainly been analyzed indirectly using auxin responsive promoters fused to reporter genes. These studies consistently found that auxin responses occur in the early dividing cells of nodule primordia, for both determinate and indeterminate nodules [145,195,203,204]. This indicates a role for auxin in cell division and differentiation, similar to their role and localization in lateral root development [24]. However, the auxin sensitivity for lateral root and nodule formation is likely different, with nodule formation requiring a low auxin response compared to lateral roots, at least in soybean [205]. Auxin responses are also evident in mature nodules, and have been localized to nodule meristems and developing vascular bundles (e.g., [195,202,204]). A recent study found additional evidence for a role of auxin in rhizobial infection and demonstrated the induction of auxin responses in infected root hairs [206]. While it remains unclear how auxin responses are directed into root hairs, the auxin responses were shown to be necessary for successful infection [206].

Computer simulations have been carried out in an attempt to understand how auxin maxima are created in a nodule primordium forming in root cortical cells. Conceptually, auxin maxima could arise from three basic mechanisms, namely increased auxin influx, decreased auxin efflux, or elevated local auxin biosynthesis. Deinum and colleagues [207] determined that the diffused and broad auxin response pattern observed during nodule primordia formation, coupled with the speed and timing with which it occurs, is most-likely contributed by a decrease in auxin export from cortical cells. A lateral relocalization of auxin efflux carriers could explain differential auxin maxima observed in the inner and middle/outer cortex of indeterminate and determinate nodule primordia, correspondingly [207].

Evidence for the occurrence of a transient auxin transport inhibition during indeterminate nodule formation can be found in multiple studies. In the model legume *M*. *truncatula* and vetch (*Vicia sativa*), radiolabelled auxin transport assays showed reduced auxin export from the site of inoculation with rhizobia within 24 h [95,146,194,208]. Furthermore, auxin response studies using a *proGH3:GUS* construct, where *GH3* is an auxin-responsive promoter, show reduced GUS staining below the sites of rhizobia infection and Nod factor application in white clover. This is consistent with results obtained from application of synthetic auxin transport inhibitors [195].

As described earlier, flavonoids have been implicated as endogenous auxin transport regulators, affecting multiple developmental programs [90]. Flavonoids are important players in nodulation control. External application of flavonoids phenocopied spatial patterns of auxin responses in *Rhizobium*-infected roots [195]. Flavonoids are induced locally during the early stages of host-*Rhizobium* interaction and could potentially act as auxin transport regulators [118]. Flavonoid-deficient *M*. *truncatula* roots with reduced expression of *CHALCONE SYNTHASE* (*CHS*), encoding the enzyme that catalyzes the first committed step in flavonoid synthesis, were unable to nodulate [95,209]. This was associated with an inability of infecting rhizobia to elicit a temporary inhibition of auxin transport [95]. Supplementation of the auxin transport inhibiting flavonol kaempferol to flavonoid-deficient *M*. *truncatula* roots reinstated nodulation ability [209]. The *compact root architecture1* (*cra1*) mutant, on the other hand, is characterized by higher than normal *CHS* activity and a lower auxin transport capacity, although this does not impede nodulation [210].

The involvement of flavonoids in auxin transport inhibition during indeterminate nodulation was supported by the finding that the cytokinin receptor mutant *cre1* of *M. truncatula*, which is defective in nodule initiation [146,211], lacks the induction of several flavonoids (naringenin, isoiquiritigenin and quercetin) in response to rhizobia [212]. The *cre1* mutant is also defective in auxin transport control and *PIN* gene induction during early stages of nodulation [146] and does not show auxin accumulation in the cortex after inoculation with rhizobia [212]. Supplementation of the *cre1* mutant with naringenin, isoliquiritigenin or quercetin restored nodulation, auxin transport control and the localization of auxin responses in dividing cortical cells, supporting the hypothesis that auxin transport inhibition by flavonoids is mediated through cytokinin signalling [212]. Interestingly, while cytokinin is also required for auxin transport control during lateral root initiation (e.g., [213,214]), this process does not appear to depend on control by flavonoids, because flavonoid deficient *Arabidopsis* and *M. truncatula* plants still form lateral roots [93,115].

The involvement of cytokinin signalling in auxin transport control and flavonoid induction is further supported by the increase of cytokinin concentrations within 3 h of Nod factor treatment in *M. truncatula* [215], which precedes auxin transport inhibition. In addition, external cytokinin application to legume roots can directly inhibit auxin transport [212] and induce flavonoid accumulation [216].

**Figure 3:**
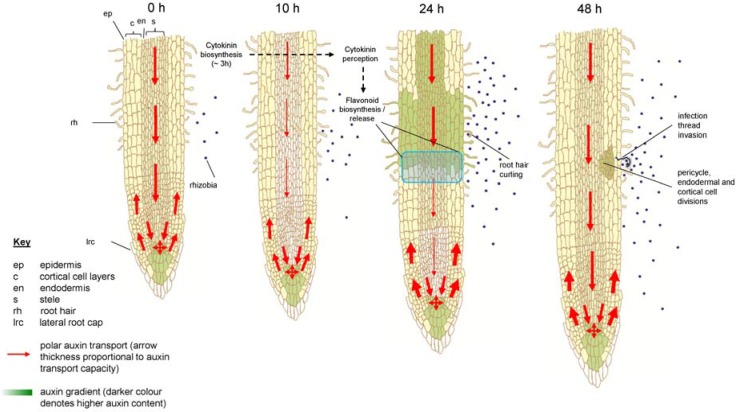
Schematic model of the regulation of auxin transport during nodulation in *Medicago truncatula*. Before rhizobia infection, auxin (indole-3-acetic acid) is transported in the acropetal direction towards the root tip. Auxin is also transported in the basipetal direction (from root tip to elongation zone) in the outer layer(s). Within 3 h after symbiosis induction (lipochitooligosaccharide treatment), cytokinin biosynthesis is upregulated in *M*. *truncatula* roots [215]. Cytokinin perception at the inner cortex induces/releases certain flavonoids, which act as inhibitors of acropetal auxin transport at the inner cortical, endodermal and/or pericycle directly underlying the rhizobia infection site [212]. Acropetal auxin transport inhibition has been observed as early as 10 h after rhizobia infection [195]. The reduction of acropetal auxin transport increases auxin concentration at the rhizobia infection site, the location of a future nodule primordium. An increase in basipetal auxin transport could also contribute to the increased auxin pool at the nodulation site [212]. Pericycle, endodermal and cortical cell divisions are activated within 48 h.

There is also evidence that auxin transport regulation by rhizobia in *M. truncatula* is under regulation by ethylene signalling. Nodulation in the ethylene insensitive *sickle* (*skl*) mutant is insensitive to the synthetic auxin transport inhibitor, 1-N-naphthylphthalamic acid (NPA) in the presence of rhizobia, suggesting that auxin transport control during nodulation requires ethylene signalling [217]. In addition, direct measurements of auxin transport in *skl* mutants showed an increase after inoculation with rhizobia compared to the wild type, accompanied by increased *MtPIN2* expression [217]. Furthermore, auxin transport inhibitors were unable to induce pseudo-nodules in the *skl* mutant [198]. Future studies could be aimed at defining the interaction between cytokinin and ethylene signalling, and the role of flavonoids in both signalling processes, for auxin transport control. A model for the control of auxin transport and accumulation in *M. truncatua* is shown in Figure 3.

In comparison to indeterminate nodulation, auxin transport inhibition has not been observed during the formation of determinate nodules, such as in soybean or *L*. *japonicus* [203,218]. Instead, an increase in acropetal polar auxin transport was observed in *L*. *japonicus* following *Rhizobium* infection [203]. Reduction in nodule numbers on isoflavonoid-deficient soybean roots was attributed to the inability of the host plant to induce Nod factor production in the symbiont *Bradyrhizobium japonicum*, rather than a host defect in isoflavonoid-mediated auxin transport inhibition [218]. While silencing of *ISOFLAVONE SYNTHASE* (*IFS*) in soybean led to increased acropetal auxin transport and auxin responses in roots, this phenotype did not prevent nodulation in a *Bradyrhizobium* strain hypersensitive to the Nod gene inducing isoflavonoid genistein [218]. Ripodas and colleagues [219] reported impaired nodulation and a reduction in auxin responses in *ISOFLAVONE REDUCTASE*-silenced roots of common bean (*Phaseolus vulgaris*), although auxin transport phenotypes were not assessed. These studies confirm a role for isoflavonoids in auxin transport and/or response in legume roots, but do not provide direct evidence for flavonoids as auxin transport regulators during nodulation in determinate nodulation programs. The difference in auxin transport control in indeterminate and determinate nodulation programs could be attributed to the different location where initial cell divisions are activated, different levels of auxin required for the initiation of cell divisions in different cell types or legume species and/or a greater requirement for lateral auxin transport in determinate nodules, which is not measured in current auxin transport assays.

### 11.3. Involvement of Auxin Transport Carriers during Legume Nodulation

Several auxin export facilitators of the PIN family have been identified in legumes. Based on sequence similarities, ten *PIN* genes were identified in *M*. *truncatula* [71], although their function has not been analyzed in detail compared to their likely *Arabidopsis* homologs. Several *PIN* genes are induced by rhizobia and Nod factor application. *MtPIN2*, *MtPIN4* and *MtPIN10* gene expression was induced within 6 h of Nod factor application to *M. truncatula* roots, while *MtPIN9* expression was strongly reduced by Nod factors [146]. Inoculation of *M. truncatula* roots with rhizobia similarly showed an induction of *MtPIN2*, *MtPIN4* and *MtPIN10* within 24 h, but no significant reduction of *MtPIN9* expression [212]. Using reporter analyses, it was found that the expression pattern of *MtPIN2* is similar to *AtPIN2* in their corresponding plants [145]. Moreover, *MtPIN2* expression during nodule initiation strongly mirrors that of lateral root initiation, supporting the hypothesis that *Rhizobium* hijacked the developmental pathway of the closely-related process of lateral root organogenesis. Knockdown of *MtPIN2*, *MtPIN3* and *MtPIN4* reduced the numbers of nodules in *M. truncatula* [145] (Table 1).

The expression of *MtPIN2* and the basipetal auxin transport in the root elongation zone were augmented after 24 h in *M*. *truncatula* in response to *Rhizobium* inoculation, but not in the non-nodulating *cre1* mutant [212]. This supports the idea that *MtPIN2* is the functional homolog of *AtPN2*, which was shown to transport auxin in basipetal direction in *Arabidopsis* [74]. An exaggerated induction of *MtPIN2* was found during nodulation of the ethylene-insensitive *skl* mutant, indicating that *MtPIN2*-mediated changes in auxin transport are linked to the higher numbers of nodules induced in this mutant [217]. The fact that *MtPIN2* is also highly expressed in root nodules suggests that MtPIN2-mediated auxin transport control is important throughout nodule development [145]. Despite this observed link between *MtPIN2* induction and increased basipetal auxin transport at the early stages on nodule development in *M. truncatula*, it is so far unknown which of the other *PIN* genes would mediate the observed inhibition of acropetal auxin transport, or possible changes in lateral auxin transport into developing nodules. It is also unknown how flavonoids or other regulators of polar auxin transport act on *PIN* gene expression and/or protein activity to exert an effect on auxin transport during nodulation, or whether other auxin transporters are targeted.

There are five auxin importers of the *LAX* family (*LIKE AUX1*) in *M*. *truncatula*. The expression of *MtLAX* genes is concentrated in dividing cells of a developing nodule and lateral root [71,148]. In the latter stages, the expression domains shifted towards the peripheral and central region of a nodule and lateral root, respectively. The authors concluded that LAX could play a role in auxin redistribution during nodule primordia development and vasculature differentiation [148]. In *L*. *japonicus*, the ABC transporter LjABCB1 was found to be highly expressed in nodules, expressed at low levels in roots and not expressed in other plant parts [149]. Localization of this transporter exclusively to uninfected cells adjacent to infected cells suggested that LjABCB1 exports auxin into symbiont-containing cells. This is reminiscent of the localization of PIN proteins in uninfected cells of *C. glauca* (see Figure 2), underlining the similarity between auxin transport into legume and actinorhizal nodules. So far, other members of the ABC transporter family have not been investigated during legume nodulation. In addition, there is a need to determine the cell specific localization of auxin transporters of different classes (see Figure 1), for example through the use of specific antibodies or GFP fusion proteins.

## 12. Auxin Acts as a Shoot-Root Regulator of Nodule Numbers

As described in the previous sections, the regulation of auxin transport at the nodule initiation site close to the root tip is crucial for the initiation of individual nodules. However, the plant also regulates the total number of nodules on the root system, and auxin transport has been linked to this systemic process as well, at least in *M. trunctula*. Nodule numbers are controlled by nitrogen availability as well as systemic regulation called autoregulation [220]. If sufficient nitrogen is available to the plant, direct uptake of inorganic nitrogen sources is preferred over the establishment of a nitrogen-fixing symbiosis. Local nitrogen availability inhibits nodulation at the infection, nodule development and nitrogen fixation stages [221]. The mechanisms of this nodulation inhibition and a possible involvement of auxin are still unclear. However, it has been clearly shown that nitrate availability regulates the related process of lateral root initiation and elongation by both local and systemic mechanisms, and that this involves changes in auxin transport and auxin signaling, suggesting that similar mechanisms might be involved in nodulation [222].

The plant also controls nodule numbers on a whole plant level by a systemic mechanism termed autoregulation of nodulation (AON), dependent on a leucine-rich repeat receptor-like kinase (NARK, NODULATION AUTOREGULATION RECEPTOR KINASE) acting in the shoot, identified in different legumes as LjHAR1 (HYPERNODULATED ABERRANT ROOT FORMATION) [223], GmNARK (NODULATION AUTOREGULATION RECEPTOR KINASE) [224] and MtSUNN [225]. An early event during nodule formation leads to the transport of a signal to the shoot, where it is perceived by NARK and causes a shoot-derived inhibitor to move back to the root system to limit further nodule formation (reviewed by [226]). The root-to-shoot signal has been identified as a peptide of the CLE (CLAVATA3/ESR-related) family [227]. The identity of the shoot-derived inhibitor is less definitive, but cytokinin and auxin transport from shoot to root have been identified as candidates in *L. japonicus* and *M. truncatula*, respectively [208,228].

The inability of the *M. truncatula* autoregulation mutant *sunn1* [225] to systemically limit nodule numbers has been linked to its inability to regulate shoot-to-root auxin transport. The *sunn1* mutant transports significantly more auxin from the shoot to the root than the wild type, and auxin concentrations in the root zone susceptible to nodule initiation are increased [208]. In addition, the auxin response gene *GH3* is expressed at much higher levels in inoculated *sunn* than in wild type roots [229]. While shoot-to-root auxin transport is inhibited by rhizobia within 24 h of inoculation in wild type roots, the approximate onset of autoregulation in *M. truncatula*, this response does not occur in the *sunn1* mutant [208]. Treatment of the shoot-root junction of *sunn1* seedlings with the auxin transport inhibitor NPA caused a reduction in nodule numbers to levels similar to the untreated wild type [208]. Consistent with these findings, expression of the auxin responsive *DR5:GFP* construct in *L. japonicus* was reduced in the autoregulation mutant *har1* and also in roots constitutively overexpression *LjCLE*, the peptide activating NARK signaling [204].

The shoot-to-root control of auxin transport during AON in *M. truncatula* is regulated independently of local auxin transport inhibition that occurs at the root tip and that is necessary for the initiation of the first nodules on the root, as the *sunn1* mutant shows local auxin transport inhibition after inoculation with rhizobia similar to the wild type, despite the difference in long distance transport [208].

The regulation of nodule numbers through AON is linked to the regulation of nodule numbers by nitrogen availability. Most AON mutants are resistant to nitrate inhibition of nodulation (e.g., [230]). The likely reason for this is that nitrate induces CLE peptides that bind to NARK in the root to inhibit nodulation locally, while rhizobia induce related CLE peptides that bind to NARK in the shoot to induce systemic autoregulation [231]. Studies in *M. truncatula* showed that if sufficient nitrate was available to the plant to inhibit nodule formation, shoot-to-root auxin transport was increased in uninoculated wild type plants, but not in the *sunn1* mutant [232]. After inoculation with rhizobia, auxin transport was reduced in plants growing under N-limiting, but not N-sufficient conditions. How the nitrogen status in the plant is “translated” into changes in shoot-to-root auxin transport remains unknown. It is possible that auxin transport control by SUNN is a more general mechanism to control root architecture in response to nitrogen, because the *sunn1* mutant is also affected in the control of lateral root density in response to nitrate through the modulation of auxin transport [232].

The NARK receptor-like kinases identified in legumes are structurally similar to *Arabidopsis* CLAVATA1 (CLV1), a receptor-kinase activated by CLAVATA3 (CLV3), also a CLV/EMBRYO SURROUNDING REGION (ESR)-RELATED PROTEIN (CLE) peptide [233]. While CLV1 is known as a regulator of shoot meristem activity, mutation of *AtCLV1* also causes excessive lateral root proliferation under N deficient conditions. Similar to control of nodule numbers by nitrate induced CLE peptides in soybean [231], N deficiency induces specific CLE peptides that bind to CLV1 in the root and inhibit the outgrowth of lateral roots in *Arabidopsis* [234]. This suggests that the AON genes may have evolved from nitrogen response genes in non-legumes that control root architecture. While this CLV1-mediated pathway for the control of lateral root numbers has so far not been linked to auxin transport, it is known that N availability alters shoot-to-root auxin transport [232,235,236], and that shoot-to-root auxin transport is important for lateral root outgrowth (e.g., [237]). We therefore hypothesize that N regulation of lateral roots in non-legumes involves a level of regulation mediated by shoot-to-root auxin transport. It is likely that the systemic regulation of nodule numbers by N through NARK has evolved from the N regulation pathways for lateral root numbers in non-legumes.

Nodule numbers are also regulated by ethylene, as demonstrated in the hypernodulation phenotype of the ethylene insensitive *skl* mutant, defective in the ethylene signaling protein ETHYLENE INSENSITIVE2 (EIN2) [238]. Whereas shoot-to-root auxin transport is similar to that in wild type in uninoculated *skl* mutants, the inhibition of auxin transport observed in wild type with rhizobia is defective in *skl* mutants [217]. The relatively increased shoot-to-root auxin transport in *skl* mutants correlates with higher numbers of nodules formed on the root, similar to the higher long-distance auxin transport in the supernodulating mutant *sunn1* [208].

Interestingly, auxin has also recently been implicated in autoregulation in actinorhizal nodulation. In Frankia-infected nodules of *C. glauca*, expression of a dominant negative form of the *C*. *glauca INDOLE-3-ACETIC ACID 7* (*CgIAA7*), which leads to inhibition of auxin responses in infected nodules, caused greater numbers of nodules to form on the whole root system [239]. This suggests that auxin accumulation in already formed nodules inhibits further nodule formation at a distance. It will be interesting to find out in the future whether this mechanism is dependent on a similar NARK-related mechanism of autoregulation as in legumes.

In AM symbioses, there is evidence that formation of mycorrhiza is autoregulated by a NARK-dependent mechanism because the systemic down-regulation of mycorrhization is repressed in the soybean NARK mutant [240]. Quantification of IAA in infected roots showed a significant increase over uninfected roots, but this response was reduced in the NARK mutant. Whether this response is due to changes in long distance auxin transport would be interesting to test.

## 13. Future Questions

Based on the studies presented, the control of auxin transport by parasitic, symbiotic and other beneficial root-associated microbes appears to be common target. A summary of auxin transporters targeted in these root–microbe interactions is shown in Table 1. Auxin plays varied roles in these different root–microbe interactions, which range from the control of root branching to the initiation of new root structures [24], but it is possible that auxin also plays a role in the control of defense responses important in infection [20,174,206]. However, so far our understanding of the exact mechanisms of auxin transport control during root–microbe interactions remains sketchy. For example, it is still unknown how flavonoids interact with the auxin transport machinery during indeterminate nodule formation, where flavonoids act, and how they are transported from the site of induction to the site of auxin transport. It is unclear why flavonoids are indispensible for auxin transport control during nodule development but not lateral root or nematode feeding structure formation [115]. In the future, examination of auxin transport mutants will be imperative for the further dissection of auxin transport control during these different processes. This could be accompanied by detailed studies on the dynamic relocalisation of auxin transporters during root–microbe interactions and a focus on the proteins known to regulate auxin transporters in *Arabidopsis*. In addition, it would be very interesting to study and compare auxin transport control in different species of legumes, and actinorhizal plants that form different types of nodules, or in legumes nodulating with β-proteobacteria, which do not require Nod factors [3]. Another interesting question remains about the common or divergent role of Nod factors and Myc factors in the regulation of auxin transport, and which other signal molecules from these symbionts are crucial for auxin transport control. While *Arabidopsis* has been a very useful model plant for the analysis of cyst nematode infection, making use of auxin transporter mutants and GFP fusion proteins, mycorrhizal and nitrogen-fixing symbioses are not formed in *Arabidopsis*. Therefore, future efforts have to focus on developing these resources in other model plants.
